# Appearance of large scavenger receptor A‐positive cells in peripheral blood: A potential risk factor for severe exacerbation of chronic obstructive pulmonary disease

**DOI:** 10.1111/pin.12776

**Published:** 2019-03-04

**Authors:** Iwao Emura, Hiroyuki Usuda, Kazuhiro Satou

**Affiliations:** ^1^ Department of Surgical Pathology Japanese Red Cross Nagaoka Hospital Nagaoka City Niigata Prefecture Japan; ^2^ Department of Internal Medicine Japanese Red Cross Nagaoka Hospital Nagaoka City Niigata Prefecture Japan

**Keywords:** chronic obstructive pulmonary disease, large scavenger receptor A‐positive cell, peripheral blood, risk factor, severe exacerbation

## Abstract

Fresh peripheral blood (PB) samples from 432 outpatients with stable chronic obstructive pulmonary disease (COPD) were examined. Patients were classified into Group A (large SRA^+^ cells were undetected) and Group B (large SRA^+^ cells were detected) and followed‐up for 1 year. Patients were further subdivided according to Global Initiative for Chronic Obstructive Lung Disease (GOLD) stage. Cox proportional hazard model had shown that Gold, Group, home oxygen therapy (HOT), and treatment were significant predictors of severe exacerbation. Six of 353 patients in Group A and 29 of 79 in Group B developed severe exacerbation. The rates of severe exacerbation were significantly higher in Group B patients, GOLD stage 2 than Group A, GOLD stage 2; in Group B, GOLD stage 3 than Group A, GOLD stage 3; and in all of Group B compared with in all of Group A. The Kaplan‐Meier curves of Group B, GOLD stages 1–4, and of all of Group B showed significantly worse rates of severe exacerbation than those of Group A, Gold 1–4, and all of Group A, respectively. The appearance of large SRA^+^ cells in the PB of patients with stable COPD may represent a useful biomarker for severe COPD exacerbation.

Chronic obstructive pulmonary disease (COPD) is characterized by periods of stability that alternate with episodes of increased severity (exacerbation) that lead to further impairment of lung function.^1^ The precise definition of exacerbation of COPD remains controversial, although, it is classified as mild, moderate, or severe.^2^ Patients experiencing severe exacerbation require hospitalization or emergency room care.^3^

Respiratory failure from these exacerbations is associated with poor health and high rates of mortality.^4^, ^5^ Therefore, sensitive biomarkers that can predict the onset of severe exacerbation are necessary, as none have yet been identified.^6^, ^7^

Chronic inflammatory changes with increased numbers of inflammatory cell types, and structural changes resulting from repeated injury and repair are found in the airway, lung parenchyma, and pulmonary vasculature of patients with COPD.^8^ We previously reported that minute lesions of alveolar damage (MLADs) were detected in lungs of patients with stable COPD.^9^ MLADs were also observed in lungs of patients with stable idiopathic pulmonary fibrosis (IPF), and acute exacerbation of IPF restrictedly occurred in patients with MLADs.^10^ Large scavenger receptor A‐positive (SRA^+^) cells were detected in peripheral blood (PB) of patients with acute exacerbation of IPF,^11^ and large SRA^+^ cells in PB might be a useful risk factor for acute exacerbation of IPF.^12^

As a result of preliminary examination, we detected large SRA^+^ cells in PB and multiple organ injury in autopsy cases with severe exacerbation of COPD. Herein, we investigated whether the presence of large SRA^+^ cells in PB of patients with stable COPD could serve as a biomarker for severe exacerbation of COPD.

## MATERIALS AND METHODS

### Patients

A total of 432 consecutive outpatients with stable COPD who visited our hospital between August 2015 and September 2017 were selected for this study (Table 1). The patients were classified into two groups: Group A (no large SRA^+^ cells detected in cytological preparations during the clinical course, *n* = 353), and Group B (one or more large SRA^+^ cells detected in cytological preparations at one or more time points during the clinical course, *n* = 79). Patients were further subdivided according to Global Initiative for Chronic Obstructive Lung Disease (GOLD) stage.^13^ Patients in Group A were followed for 1 year from the first examination of PB. Large SRA^+^ cells appeared during the clinical course in many patients in Group B. Therefore, patients in Group B were observed for 1 year from the appearance of large SRA^+^ cells to evaluate the utility of the presence of large SRA^+^ cells as a risk factor for severe exacerbation.

**Table 1:** Baseline characteristics and results of statistical analysis

	Group A (*n* = 353)	Group B (*n* = 79)	*P*	*P (log‐rank test)*
Male (*n*, %)	(272, 77%)	(72, 91%)	0.008	(−)
Age	69.5 ± 10.8	74.3 ± 9.3	<0.001	(−)
LABA, (*n*, %)	(24, 7%)	(10, 13%)	0.129	(−)
LAMA, (*n*, %)	(30, 8%)	(9, 11%)	0.552	(−)
LABA+LAMA, (*n*, %)	(38, 11%)	(17, 22%)	0.016	(−)
ICS+LABA, (*n*, %)	(25, 7%)	(7, 9%)	0.758	(−)
HOT, (%)	(10, 3%)	(15, 19%)	<0.001	(−)
Previous E. (*n*, %)	(4, 1%)	(11, 14%)	<0.001	(−)
Gold 1, (n), (S.E. *n*, % 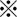 )	(129), (0, 0% 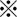 )	(16), (1, 6% 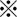 )	0.116 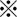	0.005 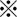
Gold 2, (n), (S.E. *n*, % 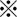 )	(160), (2, 0.01% 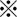 )	(30), (8, 27% 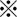 )	<0.001 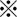	<0.001 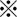
Gold 3, (n), (S.E. *n*, % 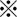 )	(52), (2, 4% 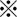 )	(27), (16, 59% 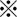 )	<0.001 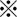	<0.001 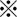
Gold 4, (n), (S.E. *n*, % 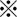 )	(12), (2, 17% 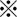 )	(6), (4, 67% 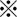 )	0.192 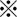	0.01 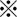
In all, (n), (S.E. *n*, % 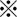 )	(353), (6, 2% 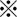 )	(79), (29, 38% 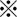 )	<0.001 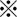	<0.001 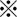

*n*, number; LABA, long‐acting beta‐2‐agonist; LAMA, long‐acting muscarinic antagonist; ICS, inhaled corticosteroid; HOT, home oxygen therapy; E, exacerbation; GOLD, Global Initiative for Chronic Obstructive Lung Disease; S.E., severe exacerbation; 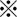, Statistical analysis of the number of patients who developed to severe exacerbation between Group A and B.

Patients whose immediate causes of hospitalization were considered to be severe bronchopneumonia, aspiration pneumonia, pulmonary embolism, acute coronary syndrome, comorbidities (such as lung cancer), heart failure, pneumothorax, or bacterial sepsis were excluded from the analysis. This study was approved by the Japanese Red Cross Nagaoka Hospital Ethical Committee (No. 2807).

### Cytology

The rest of the fresh PB samples obtained from the included patients for examination of differential white blood counts were examined (per patient: median [range], 4 [1–24]). Samples were collected in tubes containing ethylenediaminetetra‐acetic acid (EDTA) (plastic Vacutainer with K2 EDTA; Becton Dickinson, Franklin Lakes, NJ, USA). Erythrocytes were lysed with lysing reagent (826 mg NH_4_CL + 3.7 mg EDTA‐4Na + 100 mg KHCO_3_ in 100 mL H_2_O). Suspensions containing roughly 5 × 10^6^ nucleated cells in isotonic sodium chloride solution were smeared on glass slides using Auto smear CF‐12 (Sakura Seiki, Tokyo, Japan). Non‐adherent cells were gently washed off with 95% ethanol solution. Smear preparations were fixed in 95% ethanol solution and stained using the Papanicolaou method.

### Immunocytochemistry

Papanicolaou‐stained smears were used. Immunocytochemical examination was performed with Histofine Simple Stain MAX‐PO (Nichrei, Tokyo, Japan) and with diaminobenzidine as the chromogen. Mouse monoclonal anti‐human SRA was used as the primary antibody (CD204, a macrophage SRA marker, 1:200; Trans Genic, Kumamoto, Japan). Antigen retrieval was performed (using citrate buffer and microwave heating). As a negative control, phosphate‐buffered saline was added instead of the primary antibody. No positive staining was observed using this control.

## CLINICAL DIAGNOSES

### COPD

The diagnosis of COPD and grading of severity of airflow limitation were according to established international guidelines.^13^ Patients with a post‐bronchodilator forced expiratory volume % in 1 s <0.70 were classified as having COPD.

### Severe exacerbation

In the present study, hospitalized patients with increased dyspnea, sputum, and coughing and wheezing were defined as having severe exacerbation of COPD.

### Cytological diagnosis

We defined SRA^+^ cells that were over twice as large as monocytes as large SRA^+^ cells (cell diameter: >12 µ), and SRA^+^ cells smaller than large SRA^+^ cells as small SRA^+^ cells.

### Statistical analysis

All continuous variables are presented as mean ± standard deviation. Continuous variables were compared by a Wilcoxon's rank sum test. Discrete variables were analyzed using the chi‐square test or Fisher's exact test. Survival analyses were performed using the Kaplan‐Meier method and log‐rank tests. Cox proportional hazard model and stepwise selection method were performed to identify factors which affected severe exacerbation. Variables in Table 1 were considered. Gold 1, Group A and non‐treated group were used as references for Gold, Group and treated groups. In all analyses, *P*‐values <0.05 were considered statistically significant. SPSS statistics version 17.0 (SPSS Japan, Tokyo, Japan) was used for data management and analysis.

## RESULTS

### Study population, treatment and gold stages

The baseline characteristics of patients are shown in Table 1. There were significant differences in age, the number of male patients, the number of patients treated with home oxygen or a long‐acting beta‐2‐agonist and long‐acting muscarinic antagonist, and patients with previous exacerbation between Groups A and B.

### SRA^+^ cells in the systemic circulation

**Figure 1:**
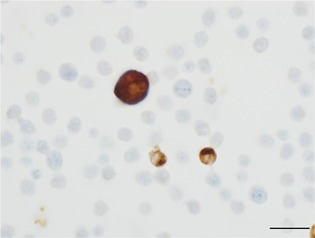
Scavenger receptor A‐positive cells in peripheral blood. Large and small scavenger receptor A‐positive cells. Immunohistochemistry for scavenger receptor A. Bar: 20 µm.

Roughly 1.0 × 10^6^ cells were smeared evenly over an area of 1.2 × 1.2 cm^2^. Small SRA^+^ cells were detected in all cases, but we could not distinguish small SRA^+^ cells from SRA^‐^ monocytes in smear preparations stained with Papanicolaou method. One to eighteen (Median [range], 4 [1–18]) large SRA^+^ cells were observed in some of the smears from patients in Group B (Fig. 1). Large SRA^+^ cells were detected one time in 54 cases and repeatedly appeared in 25 patients in Group B (per patient: median [range], 3 [2–4]; GOLD stage 1, 6 cases; GOLD stage 2, 10 cases; and GOLD stage 3, nine cases). Fifteen of the 25 patients were hospitalized more than one time during the observation period.

### Severe exacerbation and statistical analysis

**Figure 2:**
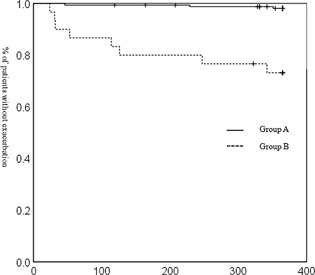
Kaplan‐Meier analysis for the development of severe exacerbation of COPD, GOLD stage 2. Kaplan‐Meier curve for severe exacerbation of COPD in Group B, GOLD stage 2 was significantly worse than that of Group A, GOLD stage 2. (*P* < 0.001, log‐rank test).

**Figure 3:**
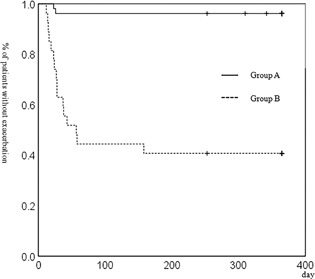
Kaplan‐Meier analysis for the development of severe exacerbation of COPD, GOLD stage 3. Kaplan‐Meier curve for severe exacerbation of COPD in Group B, GOLD stage 3 was significantly worse than that of Group A, GOLD stage 3. (*P* < 0.001, log‐rank test).

**Figure 4:**
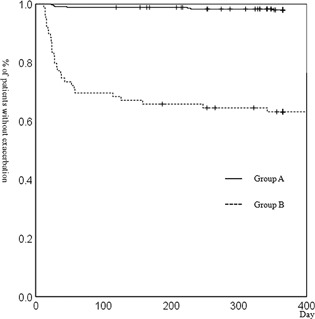
Kaplan‐Meier analysis for the development of severe exacerbation of COPD (all of Group A and B). Kaplan‐Meier curve for severe exacerbation of all of Group B was significantly worse than that of all of Group A. (*P* < 0.001, log‐rank test).

Cox proportional hazard model had shown that Gold, Group, home oxygen therapy (HOT), and treatment were significant predictors of severe exacerbation, and patients in Gold 3 or 4, Group B, HOT, treated with LABA + LAMA or ICS + LABA were susceptible to severe exacerbation, see Table 2. The rates of severe exacerbation were significantly higher in Group B, GOLD stage 2 than Group A, GOLD stage 2, in Group B, GOLD stage 3 than Group A, GOLD stage 3, and in all of Group B than in all of Group A (Table 1, all, *P* < 0.001). The Kaplan‐Meier curves of Group B, GOLD stages 1–4 and in all of Group B showed significantly worse rates of severe exacerbation compared with those of Group A, GOLD stages 1–4 and of all of Group A, (Figs. 2, 3, 4, Table 1, *P *= 0.005, <0.001, <0.001, 0.01 and <0.001, respectively, Table 1).

**Table 2:** Results of Cox proportional hazard model

	B	S.E.	*p*‐value	HR	95% CI for HR Lower‐Upper
Gold			0.014		
Gold 2	1.934	1.049	0.065	6.916	0.886–53.994
Gold 3	2.747	1.045	0.009	15.599	2.012–120.931
Gold 4	3.033	1.117	0.007	20.764	2.327–185.239
Group	2.911	0.445	<0.001	18.37	7.674–43.972
HOT	0.843	0.4	0.035	2.324	1.060–5.095
Treatment			0.001		
LABA	‐0.521	0.816	0.523	0.594	0.120–2.938
LAMA	1.103	0.567	0.052	3.013	0.991–9.154
LABA+LAMA	1.033	0.491	0.035	2.81	1.073–7.359
ICS+LABA	2.155	0.565	<0.001	8.629	2.853–26.103

GOLD, Global Initiative for Chronic Obstructive Lung Disease; HOT, home oxygen therapy; LABA, long‐acting beta‐2‐agonist; LAMA, long‐acting muscarinic antagonist; ICS, inhaled corticosteroid; B, Coefficients; S.E., standard error; HR, hazard ratio.

## DISCUSSION

According to our results, large SRA^+^ cells in PB may represent a useful biomarker for the development of severe exacerbation of COPD.

SRA antigen is expressed only on macrophages.^14^ MLADs were detected in lungs of some patients with stable COPD,^9^ and large SRA^+^ cells were observed in PB of Group B patients. SRA^‐^ monocytes differentiated into SRA^+^ cells after 5 days in culture with macrophage‐colony stimulating factor,^15^ and had differentiated into large sized macrophages by 10 days.^16^ Endothelial cells, fibroblasts, and other cells have the ability to produce one or other colony stimulating factors,^17^ and are locally activated by cells and tissues injury, which induces production and secretion of cytokines.^18^ From these findings and data, we suggest that (i) production of macrophage‐colony stimulating factor by injured endothelial cells and fibroblasts in MLADs and high concentration of macrophage‐colony stimulating factor in the PB last for long periods, and (ii) monocytes are stimulated to differentiation to large SRA^+^ cells (large sized macrophages) in PB. Consequently, large SRA^+^ cells in PB might be a useful indicator of the persistent inflammatory lung injury (MLADs) in patient with stable COPD.

We previously reported that (i) small SRA^+^ cells were detected in PB of all examined cases including healthy volunteers, (ii) multiple organ dysfunction syndrome (including acute respiratory distress syndrome) and acute exacerbation of idiopathic pulmonary fibrosis were systemic consequence with multiple organ injury induce by cytokine abnormality, (iii) large SRA^+^ cells were detected in the PB of these cases and might play important roles in the development of cytokine abnormality.^11^, ^19^ Factors that determine the survival of patients with COPD exacerbation are severity of the lung disease and extrapulmonary problems, such as ischemic heart disease, left ventricular failure, and gastro‐intestinal bleeding.^20^ As a result of preliminary examination, we detected large SRA^+^ cells in PB of autopsy cases with severe exacerbation of COPD, and multiple organs of these cases were injured. Monocytes originate from bone marrow and probably remain in circulation for no more than 36 h before migrating into the connective tissue where they increase in size, acquire multiple lysosomes, and become active in phagocytosis.^21^ These data and findings seemed to indicate that large SRA^+^ cells in PB played important roles in the development of cytokine abnormality and extrapulmonary problems in patients with COPD exacerbation. Recent study has demonstrated that activation of pattern recognition receptors initiates the activation of signaling pathways that lead to production inflammatory cytokines.^22^ It is important to investigate the pattern recognition receptors of SRA^+^ cells in the future.

Cox proportional hazard model had shown that patients in Gold 3 or 4 were susceptible to severe exacerbation. These results supported previous reports.^23^ Furthermore, our studies revealed that the rates of severe exacerbation were significantly higher in Group B, GOLD stage 2, and 3, and in all of Group B than in Group A, GOLD stage 2, and 3, and in all of Group A, respectively. The Kaplan‐Meier curves of Group B, GOLD stages 1–4 and of all of Group B showed significantly worse severe exacerbation compared with those of Group A, GOLD stage 1–4 and of all of Group A. Given these observations, we believe the appearance of large SRA^+^ cells in PB may represent a useful biomarker for severe exacerbation of COPD. Whether the appearance of these cells in PB represents a risk factor for mild or moderate exacerbation of COPD remains to be determined.

Large SRA^+^ cells repeatedly appeared in 25 patients in Group B. Fifteen of the 25 patients hospitalized more than one time during the observation period. This finding suggests that inflammatory lung injury repeatedly developed in these patients, and supports the previous observation that exacerbations accelerate the progressive decline in lung function in patients with COPD.^3^, ^24^, ^25^

This study had limitations. First, to our knowledge, few PB specimens have been examined using the Papanicolaou method. We could not identify other studies reporting the presence of large SRA^+^ cells in PB of patients with stable COPD, and could therefore not compare our results with others. Second, as this was not a multi‐center study, the patient population and standards of therapy employed may have varied from those in other centers. The results of this study may therefore not be representative of the population of COPD patients. However, the appearance of large SRA^+^ cells in PB may be because of persistent inflammatory lung injury, and may therefore serve to accurately detect disease activity or progression. Furthermore, the Papanicolaou method is used worldwide and the appearance of large SRA^+^ cells in PB is a relatively attractive marker, given that blood is readily available, and its measurement is easy to standardize. Future studies should aim to further validate the clinical utility of the presence of large SRA^+^ cells in PB as a risk factor for COPD exacerbation.

## DECLARATIONS

Ethics approval: This study was approved by the Japanese Red Cross Nagaoka Hospital Ethical committee (No. 2807).

## DISCLOSURE STATEMENT

None declared.

## AUTHOR CONTRIBUTION

IE: Conception and design of the study, acquisition and analysis of data and drafting the manuscript and figures. HU: Analysis of data and drafting the manuscript. KS: Acquisition of data and drafting the manuscript.
